# Associations of sleep pattern, sleep duration, bedtime, rising time and cardiovascular disease: Data from NHANES (2017–2020)

**DOI:** 10.1371/journal.pone.0326499

**Published:** 2025-07-11

**Authors:** Xiao Zhang, Dongling Wang, Jiangang Liu

**Affiliations:** 1 Xiyuan Hospital, China Academy of Chinese Medical Sciences, National Center for Clinical Cardiovascular Disease of Traditional Chinese Medicine, Beijing, China; 2 Graduate School, China Academy of Chinese Medical Sciences, Beijing, China; University of Modena and Reggio Emilia: Universita degli Studi di Modena e Reggio Emilia, ITALY

## Abstract

**Background:**

Sleep has a significant impact on the incidence of cardiovascular diseases (CVD), but there is no comprehensive research on this topic.

**Aims:**

Assess the association between sleep factors and the risk of cardiovascular diseases in terms of comprehensive sleep behavior.

**Methods:**

This study included 8,075 subjects from NHANES 2017–2020, excluding those with missing sleep/CVD data. Poor sleep factors: abnormal sleep duration (<7h or>8h), trouble sleeping, snoring, snort or stop breathing, sleepy during day. Each factor was scored (0–12 total), classifying sleep patterns as healthy (0–4), intermediate (5–8) or poor (9–12). Multivariable logistic regression analyzed the association between unhealthy sleep and CVD. Weighted data were used for restricted cubic spline (RCS) plots to assess the nonlinear relationship between sleep durations, bedtime, rising time and CVD.

**Results:**

Adjusted models showed significant associations between poor sleep and heart failure, myocardial infarction, stroke and hypertension (*p < 0.05*). Daytime sleepiness also increased stroke and hypertension risks (*p < 0.05*). RCS plots revealed nonlinear relationships: 7–9 hours sleep/day minimized heart failure, myocardial infarction and hypertension risks; 6–8 hours/day minimized stroke risk. Bedtime showed J-shaped and U-shaped associations with myocardial infarction and hypertension.

**Conclusion:**

This nationally representative survey revealed that poor sleep patterns, particularly sleep disorders, daytime sleepiness and reported breathing obstructions were significantly associated with an increased prevalence of cardiovascular diseases. Additionally, there was a nonlinear correlation between sleep duration, bedtime, rising time and the risk of developing cardiovascular diseases.

## Introduction

According to the 2023 WHO Health Guidelines, cardiovascular disease (CVD) remains one of the leading causes of death from non-communicable diseases globally, with approximately 17.9 million deaths attributed to CVD in 2019 [1]. CVD is the result of a combination of genetic, physiological, environmental and lifestyle factors. An indispensable approach to preventing and controlling CVD is to focus on reducing associated risk factors. Traditional modifiable risk factors include smoking, alcohol consumption and obesity. By adjusting lifestyle habits, the negative impact of all these factors on cardiovascular health can be mitigated.

Sleep is one of the important indicators reflecting overall human health. Unhealthy sleep factors, such as too little or too much sleep, sleep disorders, snoring and frequent daytime sleepiness, have been shown to have a certain correlation with CVD [2–4]. These sleep factors are often interrelated, and their combination may increase the risk of CVD [5,6]. However, most studies have examined the association between a single sleep factor and the risk of a single cardiovascular disease, without considering the intertwined relationships between multiple sleep factors and multiple cardiovascular diseases. Li found that participants in the UK Biobank with healthy sleep patterns (early sleepers, sleeping 7–8 hours per day, self-reporting no snoring, no frequent insomnia and no frequent excessive daytime sleepiness) had a lower risk of heart failure [7]. However, the study did not describe the risk of other cardiovascular diseases or provide details on specific bedtime and rising time. Another study found a significant association between late bedtime on weekdays and weekends and an increased prevalence of angina [8], but it did not explore other sleep factors or cardiovascular diseases.

Therefore, we utilized data from the National Health and Nutrition Examination Survey (NHANES) to investigate the relationship between combinations of key sleep factors characterized by a healthy sleep score and the risks of heart failure, angina pectoris, myocardial infarction, stroke and hypertension. Our aim was to assess the potential interaction between overall sleep patterns and CVD. Subsequently, we also examined the individual effects of each sleep factor on CVD to comprehensively explore the relationship between sleep and CVD.

## Methods

### Participants

**Fig 1:**
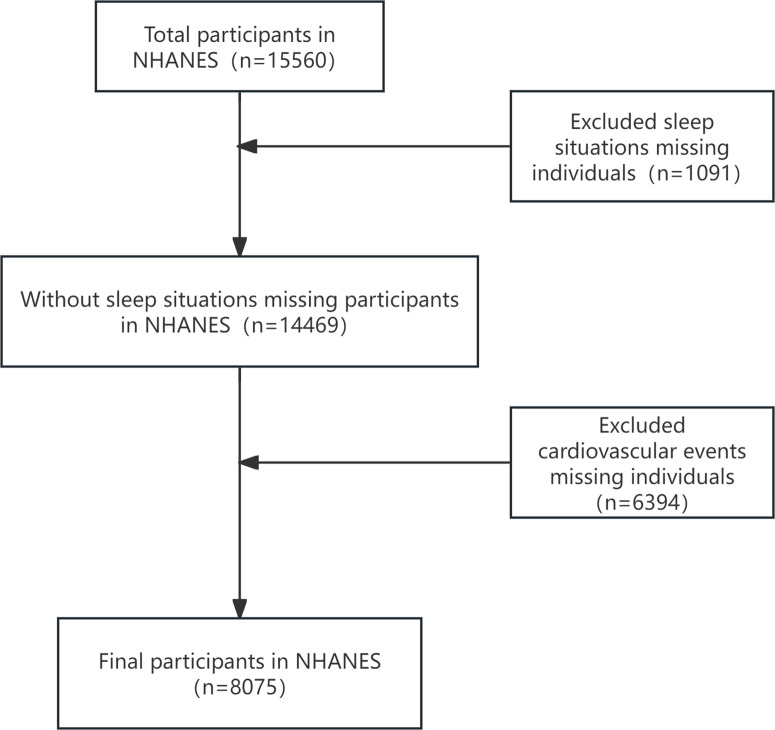
Flow diagram of the inclusion of participants.

The National Health and Nutrition Examination Survey (NHANES) is a cross-sectional study organized by the Centers for Disease Control and Prevention (CDC) in the United States. It employs a complex, multistage probability sampling method to select a representative sample of the US population every two years in order to assess the health and nutritional status of the American population. The National Center for Health Statistics Institutional Review Board approved this survey, and each participant signed an informed consent form before completing the questionnaire and undergoing laboratory tests. This study included data from 2017 to 2020, and all data were obtained from the NHANES website (https://www.cdc.gov/nchs/nhanes/index.htm). In the original NHANES survey, responses coded as “missing,” “refused,” or “don’t know” were treated as missing data. Initially, a total of 15,560 samples were included. Participants were excluded from further analysis if they (1) lacked information on sleep (such as bedtime, rising time, sleepduration and sleepduration. n = 1,091) or (2) had missing data on cardiovascular diseases (such as heart failure, angina pectoris, myocardial infarction, stroke and hypertension, n = 6,394). The final sample for analysis consisted of 8,075 participants, and the flowchart of the study sample is presented in Fig 1.

### Cardiovascular disease ascertainment

Through household interviews, participants who responded “yes” to the series of questions in the medical conditions section of the household questionnaire, such as “Has a doctor or other health professional ever told you that you have heart failure/ angina pectoris/ myocardial infarction/ stroke/ hypertension?” were considered to have the respective condition. Additionally, participants with a mean of three blood pressure measurements ≥140/90 mmHg were considered to have hypertension.

### Assessment of sleep patterns

Participants reported their sleep patterns on weekdays and rest days through self-reporting. These questions were asked in the home, by trained interviewers, using the Computer-Assisted Personal Interview (CAPI) system. We included seven aspects from the sleep habits questionnaire: bedtime, rising time, sleep duration, frequency of snoring, frequency of snort or stop breathing, trouble sleeping and sleepy during day. By calculating the bedtime, rising time and sleep duration, we obtained participants’ usual sleep patterns:

Sleep duration = (weekday sleep duration × 5 + weekend sleep duration × 2)/ 7
Bedtime = (weekday bedtime × 5 + weekend bedtime × 2)/ 7
rising time = (weekday rising time × 5 + weekend rising time × 2)/ 7

The sleep score is composed of five sleep factors: sleep duration, snoring, snort or stop breathing, trouble sleeping and sleepy during day, referencing previous relevant studies [2,7,9]. Participants who have short or long sleep durations (<7h/d or> 8h/d) [4] and have ever told a doctor about sleep disturbances receive a score of 1. The other unhealthy sleep factors are scored based on their frequency, with higher frequencies resulting in higher scores (Table 1). The total sleep score is obtained by summing the scores of the five sleep factors, ranging from 0 to 12. We categorized the overall sleep patterns into three groups based on the distribution of sleep scores: healthy sleep pattern (sleep score 0–4), intermediate sleep pattern (sleep score 5–8) and poor sleep pattern (sleep score 9–12).

**Table 1:** Sleep Pattern Scores Composition.

Sleep Pattern Scores(0 ~ 12)=Sleep Duration+Trouble Sleeping+Snoring+Snort or Stop Breathing+Sleepy During Day		
Sleep Duration	7 ~ 8h/d	0
	<7h/d or> 8h/d	1
Trouble Sleeping	No	0
	Yes	1
Snoring	Never	0
	Rarely (1 ~ 2/week)	1
	Occasionally (3 ~ 4/week)	2
	Frequently (5 or more/week)	3
Snort or Stop Breathing	Never	0
	Rarely (1 ~ 2/week)	1
	Occasionally (3 ~ 4/week)	2
	Frequently (5 or more/week)	3
Sleepy During Day	Never	0
	Rarely (1/month)	1
	Sometimes (2 ~ 4/month)	2
	Often (5 ~ 15/month)	3
	Almost always (16 ~ 30/month)	4

### Demographic variables

A number of potential confounding variables were taken into account. Age was categorized into four groups: < 30 years, 30–50 years, 50–70 years and ≥70 years. Gender was classified as male and female participants. Education level was grouped into less than 9th grade, 9–11th grade, high school graduate/ General Educational Development (GED) or equivalent, which indicates that one has completed high school education or demonstrated their knowledge level equivalent to a high school graduate through GED exam, some college or Associate Degree(AA) that means someone has started college education but has not completed the entire four-year university program or has completed a two-year associate degree program, College graduate or above. Smoking and drinking status were divided into former, current or never smokers/drinkers. Body Mass Index (BMI) was categorized into four categories: underweight (<18.5 kg/m^2^), normal weight (18.5–25 kg/m^2^), overweight (25–30 kg/m^2^) and obese (≥30 kg/m^2^). The duration of moderate-to-vigorous physical activity and sedentary time were determined based on the exercise questionnaire section.

### Statistical analyses

NHANES provides weights for each participant to account for design, non-response and stratification adjustments in order to represent the general population of the United States. Weighted population data were used to analyze the association between sleep quality, sleep duration, bedtime, rising time and the prevalence of cardiovascular diseases. Participant characteristics were compared using chi-square tests or Wilcoxon rank-sum tests. Baseline information for categorical variables was presented as unweighted frequencies and weighted percentages, while continuous variables were represented by weighted medians and quartiles. To further explore the impact of covariates on sleep and cardiovascular diseases, we employed logistic regression to establish Model 1 (roughly adjusted for age and gender) and Model 2 (fully adjusted model: age, gender, race, smoking status, drinking status and diabetes). Furthermore, weighted data were used to construct restricted cubic spline plots with 4 knots to analyze the association between different bedtimes, rising times and cardiovascular diseases. Restricted cubic spline plots approximate given data points by cubic polynomials to generate smooth curves that are smoothly connected over neighboring intervals with additional smoothness constraints to avoid sharp fluctuations in the curves. In regression analysis, restricted cubic spline plots capture the nonlinear relationship between the independent and dependent variables, and the number of nodes recommended by most researchers is 3–5 to avoid overfitting while maintaining smoothness. The R software (4.3.0) gtsummary, survey, haven, tableone, plyr, dplyr, tidyverse, arsenal and rio packages were utilized to collect and organize the data and plot the tables, the ggplot2 package was utilized to plot the restricted cubic bar graphs, and drawing forest maps, scale maps with Excel(12.1.0.17133), stata (17.0) was used to build the logistic model, *P < 0.05* was considered statistically significant for testing the research hypotheses.

### Ethics statement

The Institutional Review Board of the National Center for Health Statistics reviewed and approved the NHANES program, and all survey participants signed informed consent forms. For more information, please refer to the NHANES website (https://www.cdc.gov/nchs/nhanes/index.htm).

## Results

### The baseline characteristics of study population

**Fig 2:**
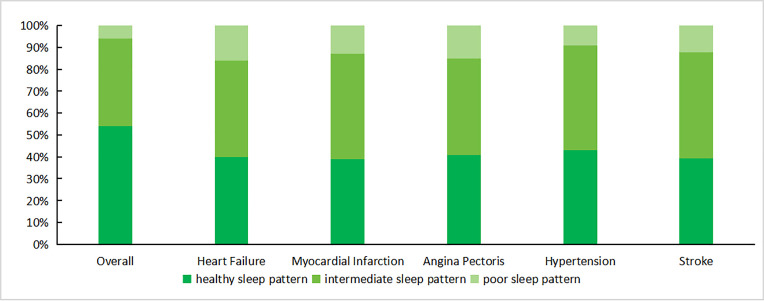
Distribution of Sleep Patterns in Cardiovascular Diseases, NHANES 2017 ~ 2020.

This research included 8075 subjects at baseline assessment, In Table 1, five cardiovascular diseases were used as outcome indicators, and factors such as age, gender, and race were examined in relation to them to summarize the characteristics of the participants in more detail. Age, race, education, smoking status, BMI and waist were significantly associated with cardiovascular diseases (heart failure, myocardial infarction, angina pectoris, hypertension and stroke), with a statistically significant higher risk of heart failure and hypertension in older adults, men, those with higher education, smoking, higher BMI and waist, longer sedentary time, poorer sleep patterns and a history of diabetes (*P < 0.05*). Regarding the risk of myocardial infarction, older people, men, higher education, smoking, alcohol consumption, higher BMI and waist, poorer sleep patterns and a history of diabetes mellitus were the high-risk groups (*P < 0.05*). Older people, higher education, smoking, alcohol consumption, higher BMI and waist, poorer sleep pattern and history of diabetes were risk factors for stroke(*P < 0.05*). Bad sleep patterns (including intermediate and poor sleep patterns) were more common among subjects with cardiovascular diseases (including heart failure, angina, myocardial infarction, stroke and hypertension) (Table 2,Fig 2).

**Table 2:** The baseline characteristics of study population.

haracteristic	Overall		Heart Failure			Myocardial Infarction			Angina Pectoris			Hypertension			Stroke		
	**N** ^***1***^	**Overall, N = 8075 (100%)** ^***2***^	**No, N = 7783 (98%)** ^**2**^	**Yes, N = 292 (2.4%)** ^**2**^	**p-value** ^***3***^	**No, N = 7729 (97%)** ^**2**^	**Yes, N = 346 (3.4%)** ^**2**^	**p-value** ^***3***^	**No, N = 7885 (98%)** ^**2**^	**Yes, N = 190 (2.1%)** ^**2**^	**p-value** ^***3***^	**No, N = 4556 (62%)** ^**2**^	**Yes, N = 3519 (38%)** ^**2**^	**p-value** ^***3***^	**No, N = 7684 (97%)** ^**2**^	**Yes, N = 391 (3.2%)** ^**2**^	**p-value** ^***3***^
**Age**	8,075	47 (33, 61)	46 (33, 61)	65 (59, 76)	<0.001	46 (32, 60)	66 (59, 74)	<0.001	46 (33, 61)	65 (55, 76)	<0.001	39 (29, 53)	59 (47, 70)	<0.001	46 (32, 61)	67 (57, 76)	<0.001
**Age**	8,075				<0.001			<0.001			<0.001			<0.001			<0.001
<50 years old		3,899 (54%)	3,871 (55%)	28 (11%)		3,872 (56%)	27 (8.1%)		3,877 (55%)	22 (14%)		3,037 (70%)	862 (29%)		3,859 (56%)	40 (11%)	
50 ~ 70 years old		2,839 (33%)	2,711 (32%)	128 (49%)		2,674 (32%)	165 (57%)		2,756 (32%)	83 (50%)		1,162 (25%)	1,677 (46%)		2,659 (32%)	180 (44%)	
70 + years		1,337 (13%)	1,201 (12%)	136 (40%)		1,183 (12%)	154 (35%)		1,252 (13%)	85 (36%)		357 (5.8%)	980 (25%)		1,166 (12%)	171 (45%)	
**Sex**	8,075				0.015			<0.001			<0.001			0.031			0.4
female		4,125 (52%)	4,001 (52%)	124 (42%)		4,022 (52%)	103 (32%)		4,045 (52%)	80 (32%)		2,406 (53%)	1,719 (49%)		3,942 (51%)	183 (55%)	
male		3,950 (48%)	3,782 (48%)	168 (58%)		3,707 (48%)	243 (68%)		3,840 (48%)	110 (68%)		2,150 (47%)	1,800 (51%)		3,742 (49%)	208 (45%)	
**Race**	8,075				<0.001			<0.001			0.014			<0.001			<0.001
Mexican American		929 (8.3%)	916 (8.4%)	13 (2.5%)		913 (8.5%)	16 (2.0%)		916 (8.4%)	13 (2.6%)		655 (10%)	274 (5.1%)		907 (8.4%)	22 (4.1%)	
Other Hispanic		812 (7.5%)	792 (7.6%)	20 (5.3%)		780 (7.6%)	32 (6.0%)		792 (7.6%)	20 (5.3%)		485 (8.0%)	327 (6.7%)		781 (7.6%)	31 (6.2%)	
Non-Hispanic White		2,813 (63%)	2,674 (63%)	139 (72%)		2,630 (63%)	183 (76%)		2,712 (63%)	101 (79%)		1,525 (62%)	1,288 (66%)		2,647 (63%)	166 (67%)	
Non-Hispanic Black		2,115 (11%)	2,020 (11%)	95 (15%)		2,037 (11%)	78 (9.3%)		2,082 (11%)	33 (5.9%)		977 (9.6%)	1,138 (14%)		1,977 (11%)	138 (16%)	
Non-Hispanic Asian		1,024 (6.1%)	1,014 (6.2%)	10 (1.9%)		1,008 (6.2%)	16 (1.9%)		1,012 (6.1%)	12 (2.8%)		690 (6.8%)	334 (4.9%)		1,009 (6.2%)	15 (2.0%)	
Other Race – Including Multi-Racial		382 (3.9%)	367 (3.9%)	15 (3.1%)		361 (3.8%)	21 (5.2%)		371 (3.9%)	11 (4.3%)		224 (3.7%)	158 (4.1%)		363 (3.9%)	19 (4.1%)	
**Education**	8,068				<0.001			<0.001			0.029			<0.001			<0.001
Less than 9th grade		582 (3.5%)	556 (3.4%)	26 (6.7%)		549 (3.4%)	33 (5.8%)		565 (3.5%)	17 (4.2%)		320 (3.3%)	262 (3.7%)		548 (3.4%)	34 (5.8%)	
9-11th grade		880 (7.0%)	835 (6.9%)	45 (10%)		820 (6.8%)	60 (13%)		846 (6.8%)	34 (15%)		448 (6.4%)	432 (7.9%)		811 (6.8%)	69 (11%)	
High school graduate/GED or equivalent		1,915 (26%)	1,825 (26%)	90 (35%)		1,832 (26%)	83 (32%)		1,873 (26%)	42 (24%)		1,005 (24%)	910 (30%)		1,796 (26%)	119 (41%)	
Some college or AA degree		2,609 (30%)	2,512 (30%)	97 (38%)		2,490 (30%)	119 (35%)		2,548 (30%)	61 (35%)		1,482 (29%)	1,127 (32%)		2,501 (30%)	108 (25%)	
College graduate or above		2,082 (33%)	2,048 (34%)	34 (9.7%)		2,031 (34%)	51 (14%)		2,047 (33%)	35 (21%)		1,298 (37%)	784 (26%)		2,021 (33%)	61 (18%)	
**Smoking status**	8,070				<0.001			<0.001			0.004			<0.001			0.007
Never smoker		4,802 (59%)	4,677 (59%)	125 (42%)		4,683 (60%)	119 (33%)		4,712 (59%)	90 (44%)		2,940 (63%)	1,862 (52%)		4,637 (59%)	165 (48%)	
Former smoker		1,875 (25%)	1,752 (25%)	123 (48%)		1,721 (25%)	154 (49%)		1,807 (25%)	68 (42%)		844 (22%)	1,031 (32%)		1,738 (25%)	137 (33%)	
Current smoker		1,393 (16%)	1,350 (16%)	43 (10%)		1,321 (16%)	72 (19%)		1,362 (16%)	31 (14%)		770 (16%)	623 (17%)		1,306 (16%)	87 (19%)	
**Alcohol intake**	7,024				0.004			<0.001			0.059			<0.001			0.016
Never drinker		646 (6.9%)	632 (6.9%)	14 (6.1%)		630 (7.0%)	16 (4.3%)		632 (6.8%)	14 (12%)		364 (6.7%)	282 (7.2%)		620 (6.8%)	26 (7.8%)	
Former drinker		1,350 (15%)	1,262 (15%)	88 (27%)		1,243 (15%)	107 (30%)		1,301 (15%)	49 (22%)		577 (12%)	773 (21%)		1,245 (15%)	105 (26%)	
Current drinker		5,028 (78%)	4,890 (78%)	138 (67%)		4,856 (78%)	172 (66%)		4,934 (78%)	94 (66%)		2,967 (82%)	2,061 (72%)		4,829 (78%)	199 (66%)	
**BMI**	7,355	29 (25, 34)	29 (25, 34)	31 (28, 36)	<0.001	29 (25, 34)	30 (27, 34)	0.001	29 (25, 34)	31 (27, 34)	0.034	27 (24, 32)	31 (27, 36)	<0.001	29 (25, 34)	31 (26, 35)	<0.001
**BMI**	7,355				<0.001			0.003			0.15			<0.001			0.031
Underweight(<18.5)		107 (1.5%)	105 (1.5%)	2 (1.0%)		105 (1.5%)	2 (1.0%)		105 (1.5%)	2 (0.9%)		87 (1.9%)	20 (0.7%)		102 (1.5%)	5 (0.8%)	
Normal(18.5 to <25)		1,770 (25%)	1,734 (25%)	36 (12%)		1,720 (25%)	50 (13%)		1,742 (25%)	28 (15%)		1,224 (31%)	546 (16%)		1,703 (25%)	67 (14%)	
Overweight(25 to <30)		2,354 (32%)	2,295 (32%)	59 (22%)		2,255 (32%)	99 (34%)		2,304 (32%)	50 (32%)		1,351 (33%)	1,003 (31%)		2,254 (32%)	100 (33%)	
Obese(30 or greater)		3,124 (42%)	2,976 (41%)	148 (64%)		2,977 (41%)	147 (52%)		3,041 (42%)	83 (52%)		1,468 (35%)	1,656 (53%)		2,961 (41%)	163 (53%)	
**Waist**	7,089	99 (89, 111)	99 (88, 111)	110 (98, 117)	<0.001	99 (88, 111)	106 (97, 116)	<0.001	99 (88, 111)	106 (96, 115)	0.001	95 (85, 106)	105 (95, 117)	<0.001	99 (88, 111)	104 (97, 115)	<0.001
**Sedentary duration**	8,062	300 (180, 480)	300 (180, 480)	360 (240, 480)	0.038	300 (180, 480)	360 (240, 540)	0.11	300 (180, 480)	300 (240, 480)	>0.9	300 (180, 480)	360 (240, 480)	<0.001	300 (180, 480)	360 (240, 480)	0.3
**Sleep pattern**	8,075				<0.001			<0.001			0.024			<0.001			<0.001
healthy sleep pattern		4,333 (53%)	4,223 (54%)	110 (40%)		4,203 (54%)	130 (39%)		4,266 (54%)	67 (41%)		2,722 (60%)	1,611 (43%)		4,174 (54%)	159 (39%)	
intermediate sleep pattern		3,190 (40%)	3,062 (40%)	128 (44%)		3,018 (40%)	172 (48%)		3,104 (40%)	86 (44%)		1,641 (36%)	1,549 (48%)		3,009 (40%)	181 (48%)	
poor sleep pattern		552 (6.2%)	498 (6.0%)	54 (16%)		508 (6.0%)	44 (13%)		515 (6.0%)	37 (15%)		193 (4.3%)	359 (9.3%)		501 (6.0%)	51 (12%)	
**Diabetes mellitus**	7,316				<0.001			<0.001			<0.001			<0.001			<0.001
Yes		1,420 (13%)	1,285 (13%)	135 (46%)		1,280 (13%)	140 (39%)		1,337 (13%)	83 (43%)		404 (6.2%)	1,016 (25%)		1,292 (13%)	128 (35%)	
No		5,896 (83%)	5,762 (84%)	134 (53%)		5,718 (84%)	178 (58%)		5,804 (84%)	92 (52%)		3,630 (90%)	2,266 (72%)		5,664 (84%)	232 (63%)	

^1^ N not Missing (unweighted).

^2^ Median (IQR); n (unweighted) (%).

^3^ Wilcoxon rank-sum test for complex survey samples; chi-squared test with Rao & Scott’s second-order correction.

### The association between sleep pattern and risk of cardiovascular diseases

**Fig 3:**
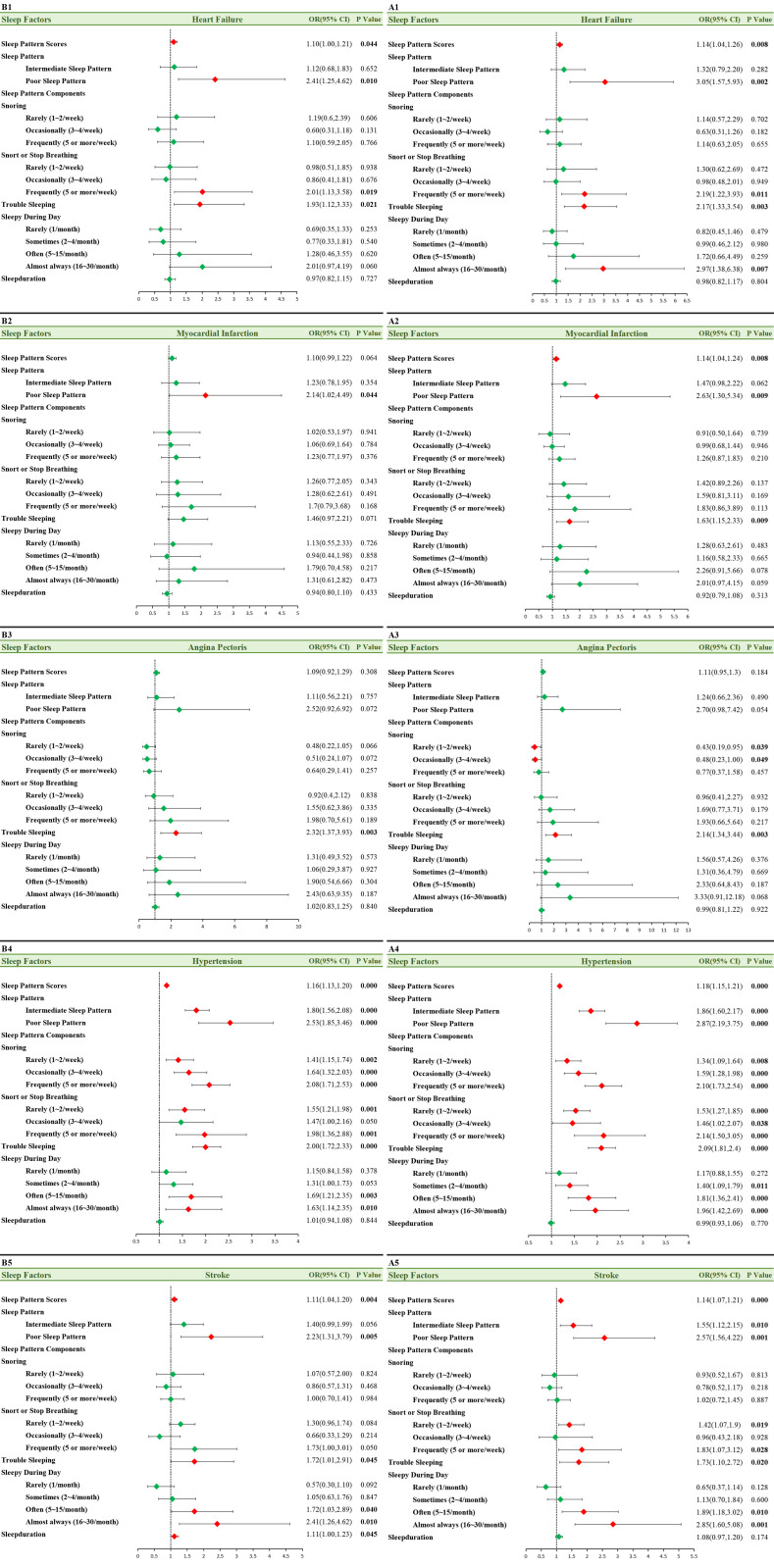
Forest Plot. The association between sleep factors and cardiovascular events (heart failure, angina pectoris, myocardial infarction, stroke and hypertension). A1 ~ A5: Model1:we adjust age and gender; B1 ~ B5: Model 2: we adjust age, gender, race, smoking status, alcohol intake, diabetes mellitus; Green box means OR value without statistical difference. Red box means OR value with statistical difference. The bars on both sides of box mean 95% CI of OR. CI, confidence intervals; OR, odds ratio.

In the model adjusted for age and gender (*Model 1, Fig 3. A1 ~ A5*), higher sleep scores were associated with higher incidences of heart failure, myocardial infarction, hypertension and stroke (*p < 0.01*). Subjects with poor sleep patterns had a higher risk of stroke and hypertension (*p < 0.05*). Compared to subjects with healthy sleep patterns, those with poor sleep patterns had a 3.05-fold and 2.63-fold higher likelihood of developing heart failure and myocardial infarction, respectively. After adjusting for potential confounding factors (*Model 2,* Fig 3*. B1, B4, B5*), sleep scores still had an impact on heart failure, hypertension and stroke (*Model 2,* Fig 3*. B1, B2, B4, B5, p < 0.05*). The impact of poorer sleep patterns on heart failure (OR: 2.41, 95% CI: 1.25–4.62), myocardial infarction (2.14(1.02,4.49)), and stroke (2.23(1.31,3.79)) remained statistically significant (*p < 0.05*). The poorer the sleep pattern, the higher the risk of hypertension, which was 1.8 and 2.53 times higher than that of people with normal sleep patterns (Fig 3*. B4, p < 0.001*).

In model 1 (Fig 3*. A1 ~ A5*), various components of sleep patterns were associated with heart failure, angina pectoris, myocardial infarction, stroke and hypertension in patients with sleep disorders (*p < 0.05*). After full adjustment in the model, these associations remained significant for heart failure, angina pectoris, stroke and hypertension (*p < 0.05*). Reported breathing obstructions was found to promote the occurrence of heart failure and hypertension. Subjects who reported daytime drowsiness exhibited a higher risk of stroke and hypertension risks, which were statistically significant in both model 1 (Fig 3*. A4 ~ A5*) and model 2 (Fig 3. B4 ~ B5) (*p < 0.05, p < 0.05*). Additionally, the frequency of snoring was positively correlated with hypertension (*p < 0.01*), while sleep duration seemed to have little association with cardiovascular diseases, with only a significant impact on stroke in model 2 (*p = 0.045*).

**Fig 4:**
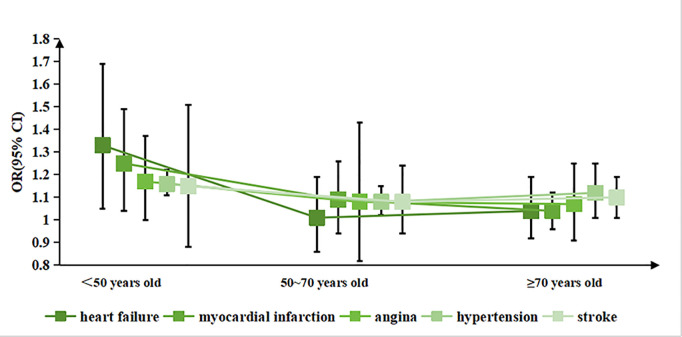
Relationship between sleep scores and cardiovascular disease based on different ages. We adjust age, gender, race, education, smoking status, alcohol intake, physical activity, sedentary duration, BMI, waist, diabetes mellitus.

We also did subgroup analyses stratified by age, adjusting for all potential confounding factors mentioned above except for the subgroup variable itself. The results of the age-stratified analysis are presented in Fig 4. In the group aged <50 years, poorer sleep scores were associated with higher risks of heart failure, myocardial infarction and hypertension. In the 50–70-year-old group, sleep scores were positively correlated with hypertension. In the ≥ 70-year-old group, poorer sleep scores contributed to the occurrence of hypertension and stroke. Across all three age subgroups, poorer sleep scores consistently increased the risk of hypertension by 1.16, 1.08 and 1.12 times, respectively (*p < 0.01*).

### The association between sleep duration and risk of cardiovascular diseases

**Fig 5:**
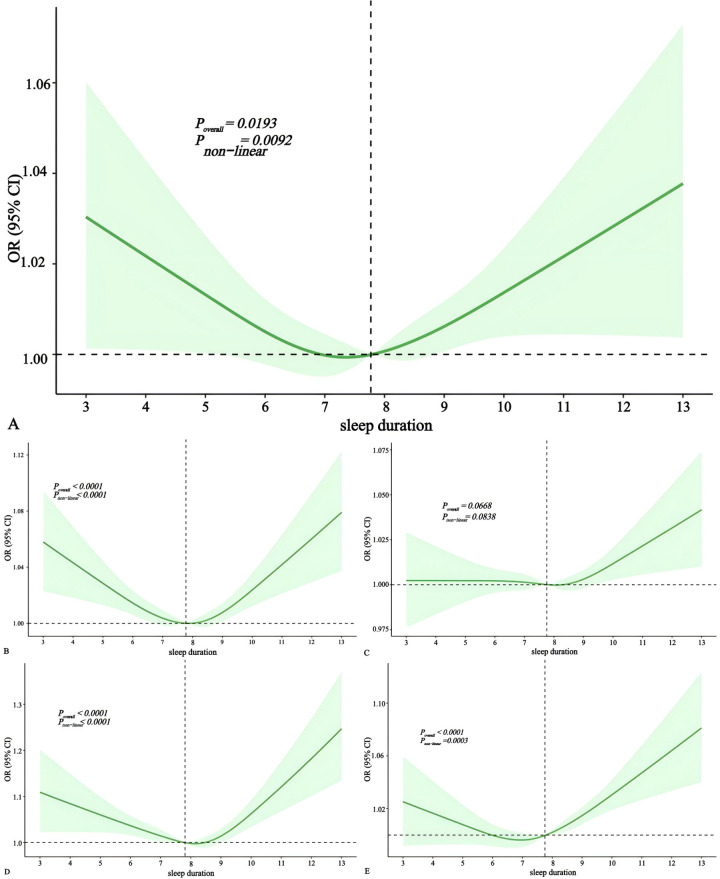
Multivariate-adjusted spline curves for sleep duration with cardiovascular diseases. The solid lines denote ORs, the dashed lines represent upper and lower limits for 95% CIs ([A] sleep duration with heart failure [B] sleep duration with myocardial infarction [C] sleep duration with angina pectoris [D] sleep duration with hypertension [E] sleep duration with stroke). ORs were derived from logistic models adjusted for age, gender, race, education, BMI, waist, smoking status, alcohol intake, physical activity, sleep status. CI, confidence interval; OR, odds ratio.

To further investigate the correlation between sleep duration and cardiovascular diseases, we subsequently plotted restricted cubic spline graphs (Fig 5). As can be seen from Fig 5 A,B, D and E, there is a nonlinear association among sleep duration and heart failure, myocardial infarction, hypertension and stroke (*p < 0.01*). Specifically, when sleep duration falls within the range of 7–9 hours per day, the risks of heart failure, myocardial infarction and hypertension are low. For stroke, the lowest risk is observed with a sleep duration of 6–8 hours per day. Both insufficient and excessive sleep durations increase the risks of heart failure, myocardial infarction and hypertension.

### The association between bedtime and risk of cardiovascular diseases

**Fig 6:**
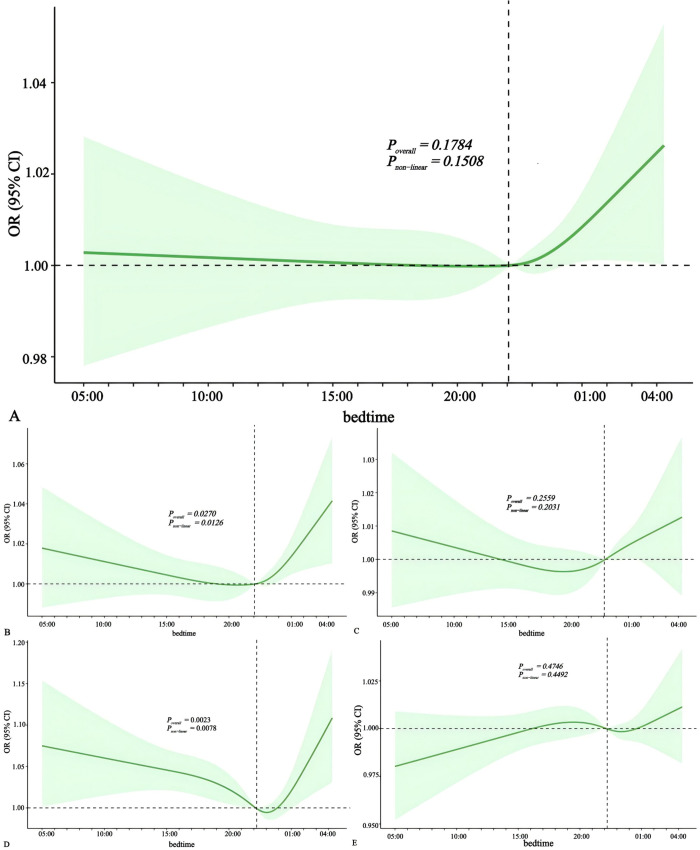
Multivariate-adjusted spline curves for bedtime with cardiovascular diseases. The solid lines denote ORs, the dashed lines represent upper and lower limits for 95% CIs ([A] bedtime with heart failure [B] bedtime with myocardial infarction [C] bedtime with angina pectoris [D] bedtime with hypertension [E] bedtime with stroke). ORs were derived from logistic models adjusted for age, gender, race, education, BMI, waist, smoking status, alcohol intake, physical activity, sleep status. CI, confidence interval; OR, odds ratio.

Based on the restricted cubic spline graphs, we observed a nonlinear relationship between bedtime and cardiovascular diseases (Fig 6). After adjusting for potential confounding factors, we found no correlation between bedtime and heart failure, angina pectoris or stroke (Fig 6A, C,E; *p > 0.05*). However, there was a J-shaped association between bedtime and myocardial infarction and a U-shaped association with hypertension (Fig 6 B,D*; p < 0.05*). Specifically, the risk of myocardial infarction was lowest when falling asleep around 20:00–21:00, while the risk of hypertension was lowest when falling asleep around 23:00.

### The association between rising time and risk of cardiovascular diseases

**Fig 7:**
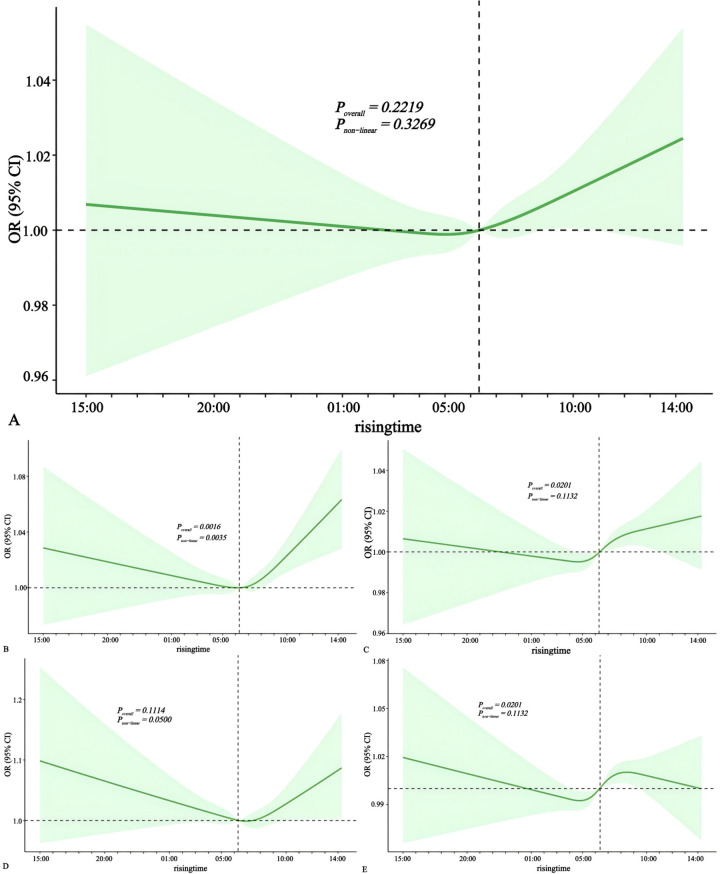
Multivariate-adjusted spline curves for rising time with cardiovascular diseases. The solid lines denote ORs, the dashed lines represent upper and lower limits for 95% CIs ([A] rising time with heart failure [B] rising time with myocardial infarction [C] rising time with angina pectoris [D] rising time with hypertension [E] rising time with stroke). ORs were derived from logistic models adjusted for age, gender, race, education, BMI, waist, smoking status, alcohol intake, physical activity, sleep status. CI, confidence interval; OR, odds ratio.

Using restricted cubic spline analysis, we examined the nonlinear relationship between wake-up time and cardiovascular diseases (Fig 7). After adjusting for potential confounding factors, we found a J-shaped association between wake-up time and myocardial infarction (Fig 7B*, p < 0.01*). The optimal wake-up time was around 6 a.m. As can be seen from the figure, waking up later had a promoting effect on the risk of myocardial infarction (*p < 0.05*).

## Discussion

Based on the NHANES, we conducted multi-level analyses of sleep-related factors such as bedtime, rising time and sleep patterns among patients with cardiovascular diseases. The results revealed that compared to healthy sleep patterns, intermediate and poor sleep patterns promoted the occurrence and development of cardiovascular diseases. Specifically, trouble sleeping were associated with heart failure, angina pectoris, myocardial infarction, stroke and hypertension. Reported breathing obstructions contributed to the occurrence of heart failure and hypertension, and a higher frequency of sleepy during day was associated with a greater risk of stroke and hypertension. Additionally, the frequency of snoring was positively correlated with hypertension risk. Logistic regression analysis showed that sleep duration had only a limited impact on stroke, while nonlinear analysis demonstrated statistically significant associations between sleep duration and heart failure, myocardial infarction, stroke and hypertension.

Further studies were conducted to investigate the relationship between bedtime, rising time and cardiovascular diseases. Restricted cubic spline (RCS) graphs were plotted, and the results indicated a J-shaped association between bedtime and myocardial infarction and a U-shaped association between bedtime and hypertension. The risks of myocardial infarction and hypertension were lowest when falling asleep around 22:00 and 23:00, respectively. Wake-up time also exhibited a J-shaped association with myocardial infarction, with the lowest risk observed when waking up around 6:00 a.m.

Sleep accounts for approximately 30% of a person’s life and is a crucial factor affecting physical health, as confirmed by various previous studies [3,10–12]. However, there is no consensus on the impact of individual sleep factors such as sleep duration, bedtime and rising time on cardiovascular diseases. Some studies suggest that both long and short sleep durations are associated with the occurrence of cardiovascular diseases [2,11], while others argue that only short sleep duration is a potential causal risk factor for several cardiovascular diseases, and that long sleep duration is not causally related to most cardiovascular diseases [13,14]. To address this, we established a logistic regression model examining the correlation between sleep duration and cardiovascular diseases. The results indicated that sleep duration only had an impact on stroke. To further investigate the nonlinear relationship between sleep duration and cardiovascular diseases, we plotted restricted cubic spline graphs. These revealed that the optimal daily sleep duration is approximately 7–9 hours, consistent with previous research [4,10]. Both shorter and longer sleep durations increase the risk of heart failure, myocardial infarction and hypertension. Sleep deprivation causes deterioration of vascular structure and function, and even one night of sleep deprivation is associated with increased arterial stiffness in healthy adults [15]. Among those ≥65 years old, long sleep duration is significantly associated with atherosclerosis [16]. In other words, both long and short sleep durations lead to endothelial damage and atherosclerosis, increasing the risk of hypertension and myocardial infarction.

Sleep apnea is an independent risk factor for the occurrence and progression of cardiovascular diseases. The prevalence of sleep apnea among cardiovascular diseases patients is as high as 40% to 80%, and the prognosis for these patients is poorer [17,18]. Patients with sleep apnea often have elevated sympathetic tone, oxidative stress, inflammatory reactions, endothelial dysfunction and metabolic disorders (such as insulin resistance and leptin resistance). These factors interact and contribute to the development and progression of cardiovascular diseases [19]. Our study also found that subjects with reported breathing obstructions had a higher risk of hypertension and heart failure, consistent with previous research. In addition, subjects who self-reported sleep disorders had a higher prevalence of cardiovascular diseases. Studies have found that mice with myocardial infarction experience a decrease in left ventricular ejection fraction and increased expression of IL-1β, IL-18, and IL-10-mRNA after sleep deprivation. This suggests that sleep disorders can increase inflammatory reactions after myocardial infarction, promoting or aggravating heart failure [20]. Another study showed that sleep deprivation reduced the synthesis of plasma exosomal miR-182-5p in mice, upregulated MYD88 in endothelial cells and activated the NF-κB/NLRP3 pathway to mediate inflammatory responses and the formation of atherosclerotic plaques. This further supports the relationship between sleep disorders and cardiovascular diseases [21]. Moreover, cardiovascular diseases can lead to inflammatory macrophage accumulation in the superior cervical ganglia (SCG), fibrosis and selective loss of pineal neuron function, resulting in reduced melatonin levels and disrupted sleep-wake cycles, further exacerbating sleep disorders and creating a vicious cycle [5,22].

To explore the association between overall sleep patterns and cardiovascular diseases, we integrated five sleep factors and established a logistic model using healthy sleep patterns as a reference. We found that subjects with imtermediate and poor sleep patterns had a higher risk of cardiovascular diseases, and this remained statistically significant after adjusting for covariates. This is similar to previous studies on the relationship between sleep patterns and cardiovascular diseases [2,23]. Therefore, there is a close relationship between sleep and the risk of cardiovascular diseases.

Subsequently, we adjusted for covariates to analyze the correlation between sleep scores and different age groups. The results revealed that sleep has a greater impact on the incidence of cardiovascular disease among people aged under 50 (*p < 0.05*). Compared to the elderly, young and middle-aged adults have a faster metabolic rate, which may result in a more significant impact of sleep on the cardiovascular system. Additionally, due to their faster work pace, irregular lifestyle and higher mental stress, young and middle-aged adults are more likely to have poor sleep habits such as insufficient sleep and staying up. In contrast, older adults often have lower homeostatic sleep pressure, exhibit less rebound sleep under conditions of sleep deprivation and report less subjective daytime sleepiness [24]. Therefore, the risk of cardiovascular disease in older adults is less affected by sleep.

To further explore the nonlinear relationship between bedtime, rising time and cardiovascular disease and to identify healthier sleep patterns, we plotted restricted cubic spline graphs. The results revealed a nonlinear correlation between bedtime and the risk of myocardial infarction and hypertension. Specifically, falling asleep around 22:00 was associated with the lowest risk of hypertension. Previous studies have also shown that late bedtime can promote the occurrence of myocardial infarction, and the underlying mechanisms may involve higher resting heart rate, blood pressure and epinephrine levels, and late bedtime can lead to increased levels of triglycerides and low-density lipoprotein and decreased levels of high-density lipoprotein, thereby increasing the risk of myocardial infarction [25]. The diurnal rhythm of blood pressure is primarily regulated by the diurnal rhythm of the sympathetic nervous system. Normal sleep reduces sympathetic activity, leading to a decrease in blood pressure. However, patients with late bedtime may experience excessive sympathetic excitation, affecting the diurnal rhythm of blood pressure [26]. Furthermore, among patients with hypertension, reduced melatonin production during the night can also affect the decrease in nocturnal blood pressure [5]. In contrast to previous studies, our findings did not show a correlation between bedtime and other cardiovascular diseases such as angina pectoris [8]. This inconsistency may be related to differences in the selection of outcome indicators and databases. The indicators chosen in this study were primarily based on self-reported survey questionnaires, while previous studies may have included indicators based on chest pain questionnaires [27]. Additionally, the data included in other studies may have originated from the UK Biobank [28], involving different ethnic and geographic groups, which may also have an impact on the correlation between sleep and cardiovascular disease.

Although an epidemiologic association between sleep and cardiovascular disease has been established, the underlying mechanisms are still under discussion. Links between sleep status and body metabolism, such as reduced glucose tolerance, sympathetic nervous system overactivity, circadian rhythms and altered hormone concentrations, are thought to play an important role in the mechanisms of cardiovascular disease. Sleep deprivation leads to increased metabolic demands to support neuronal activity during prolonged wakefulness, glycogen depletion in certain regions of the brain and activation of neuroendocrine secretion result in decreased peripheral insulin sensitivity. Subsequent stimulation of vascular smooth muscle cell proliferation and migration promotes lipid deposition and fibrosis of the arterial wall, facilitating the development of cardiovascular diseases such as hypertension, coronary heart disease and heart failure [29]. To date, there are no defined biological mechanisms to explain the association between sleep and adverse cardiovascular outcomes. However, some studies suggest that inflammation may explain the relationship between sleep and cardiovascular disease [30]. It has been found that the inflammatory cascade response occurs when awakened from sleep only once, and that multiple awakenings and sleep disruption may further exacerbate the inflammatory response [31]. Continuous stress caused by repeated snoring and upper airway closure and reopening can lead to mucosal inflammation, causing upper airway remodelling and increased connective tissue deposition in the mucosa and muscles. Intermittent hypoxia and repeated hypoxia/reoxygenation cycles induce oxidative stress, reactive oxygen species (ROS) production, activation of NF-κB, elevated expression of pro-inflammatory cytokines and chemokines, and M1-type macrophage recruitment and infiltration in different tissues, such as the vasculature and the heart, which ultimately leads to vascular remodelling, metabolic dysfunction and atherosclerosis [32]. Upper airway obstruction leads to hypoxaemia, hypercapnia and changes in intrathoracic pressure, which, together with frequent sleep arousals, cause sympathetic arousal, increased heart rate, activation of the renin-angiotensin system, and increased blood pressure [33], which in turn develops into left ventricular hypertrophy and heart failure. In addition, In addition, Sympathetic activation and hypoxia/reoxygenation cycles triggered by sleep arousal induce oxidative stress and inflammatory responses, with a consequent significant increase in activated Ca MKII levels, a significant increase in Ca MKII-dependent myocardial Na channel phosphorylation, and a dysregulation of atrial myocyte I_Na_, which means the inactivation of atrial myocyte homeostasis leads to the development of cardiac arrhythmias [34]. Suppression of inflammatory response and Ca MK II with continuous positive airway pressure ventilation may reduce sleep-induced cardiovascular risk.

### Strengths and limitations

Compared to previous studies, our research has several advantages. Firstly, we adjusted for demographic, lifestyle, and sample size factors to better represent the general population of the United States. This ensures that our findings are more applicable and relevant to a wider audience. Secondly, combined with previous studies, this study try to improve the defects of the sleep questionnaire, and analyzed the overall sleep situation to individual sleep factors. The overall sleep situation of the participants was assessed based on the sleep scores and sleep patterns, and the sleep scores consisted of five sleep factors, and the scores were made based on the frequency of the occurrence of unhealthy sleep factors, and the higher the frequency, the higher the score. Among them, bedtime, rising time, and sleep duration are calculated based on the average value of weekdays and rest days, with a total score of 0–12, and the higher the score, the poorer the sleep quality. Then we categorized the overall sleep patterns into three groups based on the distribution of sleep scores: healthy sleep pattern (sleep score 0–4), intermediate sleep pattern (sleep score 5–8) and poor sleep pattern (sleep score 9–12). We also assessed the correlation between the frequency of each sleep factor and the risk of cardiovascular disease. In our data analysis, we comprehensively investigated the relationship between various sleep factors and cardiovascular diseases. This comprehensive approach allowed us to gain a deeper understanding of the multifaceted relationship between sleep and cardiovascular health. Lastly, we conducted subgroup analyses on meaningful results, enabling us to delve deeper into specific subgroups and identify potential differences or patterns within these groups. This adds a layer of nuance and detail to our findings, making them more comprehensive and informative.

However, our study still has certain limitations. Firstly, as our research is based on a cross-sectional study from NHANES, the observed relationships cannot directly establish a causal link between sleep and cardiovascular diseases. Future studies may need to utilize longitudinal studies to validate the causal relationship. Secondly, the sleep data in our study were collected through subjective reports, which may introduce recall bias. Participants’ memories may not be entirely accurate, leading to potential inaccuracies in the data. Sleep monitoring, such as polysomnography, home sleep monitoring, or multiple sleep latency tests, is recommended for the overall assessment of sleep. However, the sleep data in the NHANES database were generated based on a sleep questionnaire, and participants mostly responded using self-reported responses, which may have recall bias. While the evaluation of sleep status has been refined, this is not a substitute for objective indicators of actual testing, and polysomnography, home sleep monitoring or multiple sleep latency tests should be performed to test sleep in future studies to obtain objective indicators for assessment. Thirdly, the outcome variables of the participants should be judged in accordance with a strict diagnostic process, although the NHANES has the collection of participants’ medication and laboratory test results, part of the data is missing, which causes certain difficulties in the diagnosis of the disease, if this part of the sample is excluded, the sample size will be greatly affected, so we chose the medical condition section of the family questionnaire, although there is recall bias, the data in this section is more complete and easier to analyze. This limits the comprehensiveness of our analysis and may have overlooked important factors that could affect the relationship between sleep and cardiovascular diseases.

## Conclusion

Poor sleep patterns, particularly sleep disorders, daytime sleepiness and reported breathing obstructions, have been significantly associated with an increased risk of cardiovascular disease. The relationship between sleep duration, bedtime and wake-up time and the risk of cardiovascular disease is nonlinear, with the optimal sleep duration ranging from 7 to 9 hours per day. Additionally, going to bed late or waking up late can both increase the risk of myocardial infarction.

## Supporting information

S1 Table The original data. (CSV)
